# In-hospital outcomes by insurance type among patients undergoing percutaneous coronary interventions for acute myocardial infarction in New South Wales public hospitals

**DOI:** 10.1186/s12939-023-02030-1

**Published:** 2023-10-23

**Authors:** Juliana de Oliveira Costa, Sallie-Anne Pearson, David Brieger, Sanja Lujic, Md Shajedur Rahman Shawon, Louisa R. Jorm, Kees van Gool, Michael O. Falster

**Affiliations:** 1 Medicines Intelligence Research Program, School of Population Health – Faculty of Medicine and Health, UNSW Sydney, Sydney, Australia; 2 Centre for Big Data Research in Health – Faculty of Medicine and Health, UNSW Sydney, Sydney, Australia; 3 Concord Clinical School – The University of Sydney, Sydney, Australia; 4 Centre for Health Economics Research and Evaluation - University of Technology Sydney, Sydney, Australia

**Keywords:** Healthcare disparities, Health insurance, Hospitals, Myocardial infarction, Percutaneous coronary intervention

## Abstract

**Background:**

International evidence suggests patients receiving cardiac interventions experience differential outcomes by their insurance status. We investigated outcomes of in-hospital care according to insurance status among patients admitted in public hospitals with acute myocardial infarction (AMI) undergoing percutaneous coronary intervention (PCI).

**Methods:**

We conducted a cohort study within the Australian universal health care system with supplemental private insurance. Using linked hospital and mortality data, we included patients aged 18 + years admitted to New South Wales public hospitals with AMI and undergoing their first PCI from 2017–2020. We measured hospital-acquired complications (HACs), length of stay (LOS) and in-hospital mortality among propensity score-matched private and publicly funded patients. Matching was based on socio-demographic, clinical, admission and hospital-related factors.

**Results:**

Of 18,237 inpatients, 30.0% were privately funded. In the propensity-matched cohort (*n* = 10,630), private patients had lower rates of in-hospital mortality than public patients (odds ratio: 0.59, 95% CI: 0.45–0.77; approximately 11 deaths avoided per 1,000 people undergoing PCI procedures). Mortality differences were mostly driven by STEMI patients and those from major cities. There were no significant differences in rates of HACs or average LOS in private, compared to public, patients.

**Conclusion:**

Our findings suggest patients undergoing PCI in Australian public hospitals with private health insurance experience lower in-hospital mortality compared with their publicly insured counterparts, but in-hospital complications are not related to patient health insurance status. Our findings are likely due to unmeasured confounding of broader patient selection, socioeconomic differences and pathways of care (e.g. access to emergency and ambulatory care; delays in treatment) that should be investigated to improve equity in health outcomes.

**Supplementary Information:**

The online version contains supplementary material available at 10.1186/s12939-023-02030-1.

## Introduction

Cardiovascular disease (CVD) is the leading cause of mortality and disability worldwide [1]. In Australia, over 590,000 hospital admissions are for CVD each year, with acute myocardial infarction (AMI) contributing to more than 10% of CVD hospitalisations and over 17% of CVD deaths [2]. Reducing major in-hospital cardiovascular outcomes and deaths is a focus of the Australian national health strategy [3]. A large proportion of CVD hospitalisations and deaths are avoidable through timely detection and increased access to effective preventative and treatment, including access to coronary artery bypass grafting (CABG) and percutaneous coronary intervention (PCI). PCI is the preferred therapy for symptomatic patients presenting with AMI and is the dominant revascularisation procedure in Australia and internationally [2, 4–6].

Despite advances in treatment and outcomes, socioeconomic inequities in access to and outcomes following cardiac care remain [2, 7–11]. In countries with highly privatised health systems, AMI inpatients with health insurance, compared with uninsured patients, have higher rates of revascularisation [12] and better outcomes such as shorter length of stay [12] and 30%-40% lower rates of in-hospital mortality [12–14], even after adjusting for patient demographic, socioeconomic and clinical characteristics. These differences likely reflect disparities in patient access to surveillance, preventive and hospital care, as well as physician preferences and incentives in performing procedures based on patient insurance status [7, 10, 15].

Evidence of socioeconomic disparities in CVD hospital care are also emerging from countries with universal health systems [7, 10, 16, 17]. In Australia, private health insurance supplements publicly funded universal health care; with almost half of Australians having private health insurance. Private insurance is associated with higher socioeconomic and better health status [18]. Privately insured patients experience shorter waiting times for elective surgery, can choose their treating physician and access private hospitals [19, 20]. As such, privately insured patients have greater access to cardiac interventions, including angiography, angioplasty, stenting, CABG and catheter ablation at the time of presentation [11, 21, 22].

The majority of emergency care in Australia, however, occurs within public hospitals, which account for over 80% of emergency AMI hospitalisations [11]. Patients in public hospitals with private health insurance can elect to be treated as a private or public patient [19]. In recent years, the number of privately funded public hospital admissions has increased, [23] raising concerns about the equity of care provided [24]. However, there is limited evidence on disparities in care outcomes between public and private CVD patients treated in Australian public hospitals [21, 25].

We investigated if private health insurance is associated with better in-hospital outcomes of care among patients in Australian public hospitals admitted with AMI undergoing PCI, the most common revascularisation procedure. Specifically, we measured hospital acquired complications (HACs), hospital length of stay (LOS) and in-hospital mortality among people undergoing their first-observed PCI.

## Methods

### Setting and dataset

New South Wales (NSW) is Australia’s most populous state, with a population of approximately 8 million people. We used de-identified, linked data from the NSW Admitted Patient Data Collection (APDC), NSW Emergency Department Data Collection (EDDC) and the Registry of Births, Deaths and Marriages (RBDM) Death Registrations data. The APDC is a census of all inpatient separations (discharges, transfers, and deaths) from public and private hospitals, public psychiatric hospitals, multi-purpose services, and private day procedure centres. The APDC contains data relating to patient demographics (e.g. age, sex, area of residence); admission characteristics (e.g. funding source, emergency/elective status, admission and separation date); patient diagnoses (condition onset flag; diagnoses coded according to the International Classification of Diseases 10^th^ Revision Australian Modification [ICD-10-AM]); and procedures (coded according to the Australian Classification of Health Interventions [ACHI]). The EDDC data records characteristics of presentations to the emergency departments of public hospitals (e.g. visit type, mode of arrival, triage category). The RBDM Death Registrations data contain the date of death.

The study dataset included all patients who had any procedure recorded within this time period. Data linkage was performed by the NSW Centre for Health Record Linkage (CHeReL) as part of its Master Linkage Key.

### Study population

We included people aged 18 years or older who had a primary diagnosis of AMI and underwent first-observed (index) PCI procedure between 1st January 2017 to 31st December 2020 (See Supplementary Box 1 for codes and definitions). Where patients had multiple records for an admission (e.g. transfer between facilities; change from acute to sub-acute care) we treated these as a continuation of the same admission.

We excluded patients with a PCI procedure in the prior five years, as well as patients with a hybrid revascularisation (i.e. receiving both PCI and CABG) in their index admission, given these patients may differ from those eligible for PCI alone [26]. We also excluded patients with inconsistent records suggesting possible data linkage errors (e.g. APDC records after the date of death).

We defined patients as being privately insured if any of the episodes of care within an admission were billed as a private patient (exposure group), or as publicly insured if all episodes of care were billed as a public patient (comparison group). We excluded patients insured via other schemes (i.e. veterans, workers compensation) on the index admission.

### Patient, admission and hospital characteristics

We measured factors potentially related to patients’ use of private health insurance and outcomes of care, including their demographics, clinical characteristics, characteristics of the index admission, and the hospital of admission and procedure. Patient demographics included age, sex, as well as Index of Relative Socioeconomic Disadvantage (IRSD) and remoteness of the area of the patient’s area (Statistical Area 2) of residence [27, 28]. Clinical characteristics of the index admission included the type of AMI (ST-elevated myocardial infarction [STEMI], non–ST-elevated myocardial infarction [NSTEMI], or unspecified) and type of PCI procedure (multi-vessel; multi-stent).

We measured patient morbidities within the index admissions or in the 12 months prior, including the Charlson Comorbidity score [29] and individual diagnoses (e.g. hypertension, diabetes, renal disease). We measured history of smoking using diagnoses within the index admission and the five years prior [30]. We also measured history of CABG, prior admissions for AMI or stroke, and number of hospital admissions and emergency department (ED) presentations prior to the index admission. Admission characteristics included the emergency status on admission and the mode of arrival for those admitted via the ED. Further details on the definition of variables are in Supplementary Table 1.

We identified the peer group of the hospital of admission, categorised as major, large, district or other hospitals (See supplementary Table 2). We measured hospital volume of procedures, using a data-driven approach to calculate quartiles of annual number of PCIs performed within each hospital. Volume was measured for the hospital performing the PCI procedure, noting that some private patients may have been transferred to a private facility for the PCI during their public hospital stay.

### Propensity-matched cohort

To control for potential confounding by differences at the index admission, we used propensity scores to match private and publicly funded patients with similar demographic and clinical characteristics, as well as peer group of the hospital of admission and volume of procedures of the hospital undertaking the PCI. We used multivariate logistic regression to estimate the propensity score, using the variables described in Table 1). We matched patients using the 1:1 nearest neighbour without replacement (i.e. private and public patients are paired only once) and allowing the selection of a match within 0.2 propensity score standard deviation. We assessed adequate balance of the baseline covariates using standardised mean differences with a threshold of 10% [31].

**Table 1:** Socio-demographic, clinical, admission and hospital-related characteristics according to private health insurance status before and after propensity score matching (New South Wales, 2017–2020)

Characteristics	Unmatched cohort			Matched cohort		
	**Private**	**Public**	***p*-value**	**Private**	**Public**	***p*-value**
**Total number of patients (N)**	5486	12,751		5315	5315	
Sex:			0.008			0.856
Male	4171 (76.0)	9455 (74.2)		4034 (75.9)	4025 (75.7)	
Female	1315 (24.0)	3296 (25.8)		1281 (24.1)	1290 (24.3)	
Mean age (SD)	68.0 (12.3)	64.3 (13.0)	< 0.001	67.8 (12.3)	67.8 (12.3)	0.739
Age group:			< 0.001			0.869
18–40 years	59 (1.08)	331 (2.60)		59 (1.1)	61 (1.2)	
41–50 years	367 (6.69)	1480 (11.6)		367 (6.9)	360 (6.8)	
51–60 years	1019 (18.6)	3160 (24.8)		1009 (19.0)	1034 (19.5)	
61–70 years	1571 (28.6)	3375 (26.5)		1532 (28.8)	1499 (28.2)	
71–80 years	1458 (26.6)	2685 (21.1)		1397 (26.3)	1370 (25.8)	
81 + years	1012 (18.4)	1720 (13.5)		951 (17.9)	991 (18.6)	
Type of AMI:			< 0.001			0.925
NSTEMI	3075 (56.1)	6691 (52.5)		2953 (55.6)	2968 (55.8)	
STEMI	2376 (43.3)	5963 (46.8)		2327 (43.8)	2310 (43.5)	
Not specified	35 (0.64)	97 (0.76)		35 (0.7)	37 (0.7)	
Multi vessel PCI	934 (17.0)	1928 (15.1)	0.001	894 (16.8)	886 (16.7)	0.856
Multi stent PCI	1908 (34.8)	4031 (31.6)	< 0.001	1836 (34.5)	1853 (34.9)	0.744
Charlson Comorbidity Score:			0.001			0.480
1–2 comorbidities	4883 (89.0)	11,124 (87.2)		4730 (89.0)	4706 (88.5)	
3 or more comorbidities	603 (11.0)	1627 (12.8)		585 (11.0)	609 (11.5)	
Charlson score of comorbidities, mean (SD)	1.49 (0.87)	1.56 (0.92)	< 0.001	1.49 (0.87)	1.51 (0.89)	0.262
Current smoking	1028 (18.7)	5097 (40.0)	< 0.001	1026 (19.3)	1047 (19.7)	0.624
Hospital admissions in the prior year:			< 0.001			0.200
None	3530 (64.3)	9111 (71.5)		3496 (65.8)	3583 (67.4)	
1–2	1450 (26.4)	2860 (22.4)		1373 (25.8)	1304 (24.5)	
3 or more	506 (9.2)	780 (6.12)		446 (8.4)	428 (8.1)	
ED presentations in the prior 3 months:			< 0.001			0.769
None	209 (3.8)	665 (5.2)		205 (3.9)	218 (4.1)	
1	4719 (86.0)	10,359 (81.2)		4560 (85.8)	4538 (85.4)	
2 or more	558 (10.2)	1727 (13.5)		550 (10.3)	559 (10.5)	
ED presentations in the prior year:			< 0.001			0.769
None	262 (4.8)	849 (6.7)		258 (4.9)	280 (5.7)	
1	3921 (71.5)	8064 (63.2)		3780 (71.1)	3759 (70.7)	
2 or more	1303 (23.8)	3838 (30.1)		1277 (24.0)	1276 (24.0)	
Prior stroke	33 (0.6)	56 (0.4)	0.185	31 (0.6)	33 (0.6)	0.900
Prior AMI	119 (2.2)	364 (2.9)	0.009	116 (2.2)	137 (2.6)	0.203
Prior CABG	15 (0.3)	40 (0.3)	0.758	15 (0.3)	22 (0.4)	0.323
Heart failure	519 (9.5)	1288 (10.1)	0.193	505 (9.5)	554 (10.4)	0.120
Cardiac Arrhythmia	1088 (19.8)	2258 (17.7)	0.001	1042 (19.6)	1040 (19.6)	0.981
Cardiogenic shock	90 (1.6)	282 (2.2)	0.014	89 (1.7)	86 (1.6)	0.879
Cardiac arrest	61 (1.1)	171 (1.3)	0.232	60 (1.1)	68 (1.3)	0.534
Peripheral vascular disease	46 (0.8)	91 (0.7)	0.423	45 (0.9)	46 (0.9)	1.000
Hypertension	3305 (60.2)	7220 (56.6)	< 0.001	3182 (59.9)	3183 (59.9)	1.000
Diabetes	1071 (19.5)	3061 (24.0)	< 0.001	1054 (19.8)	1061 (20.0)	0.884
Coagulopathy	62 (1.1)	148 (1.2)	0.919	61 (1.2)	61 (1.2)	1.000
Pulmonary disease	120 (2.2)	493 (3.9)	< 0.001	120 (2.3)	122 (2.3)	0.948
Renal disease	282 (5.1)	747 (5.9)	0.058	276 (5.2)	287 (5.4)	0.665
Remoteness:			< 0.001			0.926
Major cities	4099 (74.7)	9093 (71.3)		3946 (74.2)	3942 (74.2)	
Inner regional	1090 (19.9)	2717 (21.3)		1073 (20.2)	1075 (20.2)	
Outer regional	202 (3.7)	672 (5.3)		201 (3.8)	193 (3.6)	
Remote/ very remote	10 (0.2)	62 (0.5)		10 (0.2)	9 (0.2)	
Other	85 (1.5)	207 (1.6)		85 (1.6)	96 (1.8)	
Socioeconomic status*			< 0.001			0.348
1st quintile—Most disadvantaged	909 (16.6)	4232 (33.2)		909 (17.1)	898 (16.9)	
2nd quintile	1189 (21.7)	3297 (25.9)		1183 (22.3)	1213 (22.8)	
3rd quintile	1079 (19.7)	2194 (17.2)		1067 (20.1)	1125 (21.2)	
4th quintile	952 (17.4)	1428 (11.2)		911 (17.1)	904 (17.0)	
5th quintile—Least disadvantaged	1270 (23.1)	1389 (10.9)		1158 (21.8)	1077 (20.3)	
Other	87 (1.59)	211 (1.65)		87 (1.6)	98 (1.8)	
Emergency status on admission			< 0.001			0.348
Elective/ Not assigned	261 (4.7)	871 (6.8)		260 (4.9)	277 (5.2)	
Emergency	5225 (95.2)	11,880 (93.2)		5055 (95.1)	5038 (94.8)	
ED mode of arrival			< 0.001			0.500
Emergency services	2870 (52.3)	6337 (49.7)		2779 (52.3)	2734 (51.4)	
Other	2332 (42.5)	5526 (43.3)		2258 (42.5)	2280 (42.9)	
Unknown/NA	284 (5.2)	888 (7.0)		278 (5.2)	301 (5.7)	
Hospital of admission peer group:			< 0.001			0.776
Principal referral	3651 (66.6)	7718 (60.5)		3509 (66.0)	3491 (65.7)	
Major hospitals	1446 (26.4)	4278 (33.6)		1432 (26.9)	1470 (27.7)	
District hospitals	300 (5.5)	637 (5.0)		296 (5.6)	279 (5.3)	
Other	89 (1.6)	118 (0.9)		78 (1.5)	75 (1.4)	
Hospital annual volume of PCI procedures:			< 0.001			0.820
1st quartile	1286 (23.4)	3450 (27.1)		1268 (23.9)	1299 (24.4)	
2nd quartile	1438 (26.2)	2856 (22.4)		1371 (25.8)	1343 (25.3)	
3rd quartile	1665 (30.3)	3351 (26.3)		1595 (30.0)	1575 (29.6)	
4th quartile	1097 (20.0)	3094 (24.3)		1081 (20.3)	1098 (20.7)	
Year of admission:			0.004			0.998
2017	1317 (24.0)	2895 (22.7)		1276 (24.0)	1269 (23.9)	
2018	1450 (26.4)	3240 (25.4)		1387 (26.1)	1389 (26.1)	
2019	1454 (26.5)	3374 (26.5)		1414 (26.6)	1413 (26.6)	
2020	1265 (23.1)	3242 (25.4)		1238 (23.3)	1244 (23.4)	

*ED* Emergency department, *PCI* Percutaneous coronary interventions, *CABG* Coronary artery bypass grafting procedure,*Based on the *IRSD* Index of relative socioeconomic disadvantage, *NA* Not applicable

### In-hospital outcomes

For each patient in our matched cohort, we estimated the prevalence of HACs, the total hospital length of stay (LOS), and all-cause in-hospital mortality. We measured HACs as the occurrence of any hospital-acquired complications within the admission, as per the indicators developed by the Australian Commission on Safety and Quality in Health Care [32]. The HAC indicator includes various types of complications, such as cardiovascular complications, healthcare associated infections, surgical complications requiring an unplanned return to theatre, respiratory complications, and delirium. We calculated total hospital LOS as the difference between the admission end and start dates (including transfers and type change separations). For same-day admissions, we assigned LOS of one day. We identified in-hospital mortality if either the mode of separation for any episode of care within the admission was death, or if the patient had a death date registered in the RBDM Death Registrations between the admission start and end date.

### Statistical analysis

We conducted a descriptive analysis of patient characteristics according to insurance status in both non-matched and propensity-matched cohorts. We presented categorical variables as proportions and quantitative variables by the mean or median values and respective standard deviation (SD) or Interquartile range (IQR). All other statistical analysis were performed in our propensity-matched cohort, only.

We conducted a bivariate analysis by private health insurance status to calculate average HACs, hospital length of stay, and in-hospital mortality rates. We estimated the average LOS as the sum of the LOS of all patients divided by the total number of patients. We excluded patients with outlying LOS from these analyses, using a threshold based on LOS distribution [Outlier bound = 75^th^ centile + 10 * (75^th^—25^th^ centile)] [33].

To assess the association between private health insurance status and outcomes, we calculated odds ratios (ORs) for in-hospital mortality and HACs using Wald tests to test statistical differences between groups. We reported the reduced risk of in-hospital death using the formula: PR private – PR public patients, where PR is the Prevalence Ratio of in-hospital mortality in each group. We estimated average LOS differences using ordinary least squares regression with gauss family given the coefficient from this model is directly interpreted as differences in average LOS between groups. We used public patients as the referent group. We used cluster-robust standard errors in these analyses to account for the matched nature of the sample [31].

We performed cohort selection and data manipulation using SAS V.9.4 (SAS Institute Inc., Cary, NC, USA). We used "MatchIt" and “Cobalt” packages to conduct propensity score matching, diagnostics and estimate treatment effects, as well as conducted the remaining statistical analysis using R V.4.1.1 (R Core Team 2021, Vienna, Austria). For all statistical analyses, we considered the level of significance of 0.05 using two-sided tests.

### Post-hoc analyses

We conducted a series of mutually exclusive post-hoc analyses to assess the robustness of our mortality findings. First, we accounted for differing propensity to be transferred between hospitals (e.g. for day procedure in a private facility), which may impact access to services and delays in decision-making processes between public and private patients [33]. We excluded patients transferred to other hospitals (public or private) for their PCI procedure, identified if the hospital performing the procedure differed from the hospital of admission.

Second, as there may be further socioeconomic differences between patients who do and do not hold private health insurance than those measured in our study, we investigated reported insurance status during the hospital admission (noting not all patients with health insurance will report so during the admission). To assess the potential further confounding effects by socioeconomic status, we: (i) restricted to patients recorded as holding private health insurance within the hospital admission, and (ii) restricted to patients where reported insurance status was in agreement with their billing status.

Third, we varied our mortality definition to account for differences in follow-up time and discharge protocols between public and privately treated patients (e.g. to private rehabilitation facilities). We (i) restricted the assessment window to 30 days following the PCI episode and (ii) increased the assessment window to include both in-hospital mortality and 30 days following discharge.

Fourth, we stratified our analyses by AMI type (STEMI or NSTEMI/ non-specified AMI), given differences in severity of the admission and that these patients may have different pathways of care, and by remoteness, given there are differences in access to care across areas.

Finally, we estimated the impact of unmeasured confounding in our analysis using the E-value [34]. This measure indicates the minimum strength of association that an unmeasured confounder would need to have to fully explain away the observed association.

## Results

**Fig. 1:**
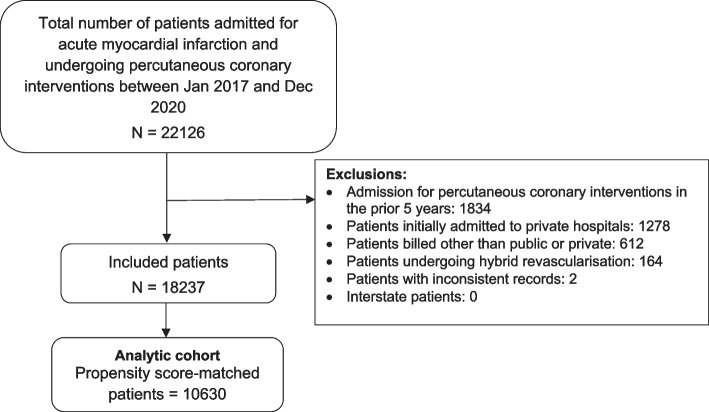
Patient selection for propensity score matched analyses

We identified 18,237 patients admitted to New South Wales public hospitals with AMI and undergoing PCI meeting our eligibility criteria, of which 5,486 (30.1%) were private patients. A total of 5,315 private patients were propensity-score matched to a public patient, leading to 10,630 patients for analysis (Fig. 1).

A summary of the index admission characteristics for the unmatched and matched cohorts is shown in Table 1. Prior to matching, private patients were more likely to be male, slightly older, a slightly higher proportion of NSTEMI patients, and a higher proportion having multi-vessel or multi-stent PCI than public patients. Private patients also had better health at the index admission than public patients, with a lower prevalence of several morbidities (e.g., heart failure, peripheral vascular disease, diabetes, chronic pulmonary disease, cardiogenic shock), a lower comorbidity score, a lower proportion being current smokers and lower proportion of multiple ED presentations. Private patients had higher socioeconomic status and were living more often in major cities than public patients, were more likely to be admitted for urgent care, arrive in the ED using emergency services, be admitted at larger hospitals and receive PCI procedures more often at hospitals with intermediate levels of annual procedures (Table 1).

After matching, socio-demographic, clinical, admission or hospital-related characteristics at the index admission were balanced between private and public patients, evidenced by standardised mean differences below 10% in the distribution of their characteristics (Supplementary Fig. 1).

### In-hospital outcomes in the propensity-matched cohorts

Hospital Acquired Complications: The occurrence of at least one HAC within the admission was relatively common (*n* = 802, 7.5%). The five most common HACs were cardiac complications (*n* = 251, 2.4%), healthcare associated infection (*n* = 240, 2.3%), surgical complications (*n* = 190, 1.8%), delirium (*n* = 116, 1.1%) and respiratory complications (*n* = 112, 1.1%). There were no clinical meaningful differences between private compared to public patients in rates of HACs during the admission (OR: 0.97, 95%CI: 0.84, 1.12) (Tables 2. and 3).

**Table 2.** In-hospital outcomes in the propensity-matched cohorts according to private health insurance status (New South Wales, 2017–2020)

	**Private patients**	**Public patients**	***p*-value**
	***N* = 5315**	***N* = 5315**	
**Hospital acquired complications (any)**			
Any within admission (%)	395 (7.4%)	407 (7.7%)	
Odds Ratio (95% CI)	0.97 (0.84, 1.12)	Ref	0.686
**Length of stay (LOS)**^**a**^			
Average LOS in days (SD)	5.11 (± 4.40)	5.22 (± 4.39)	
Mean difference (95% CI)	-0.11 (-0.28, 0.05)	Ref	0.182
**In-hospital mortality**			
In-hospital mortality (%)	86 (1.6%)	143 (2.7%)	
Odds Ratio (95% CI)	0.59 (0.45, 0.77)	Ref	< 0.001

^a^Excluded 74 outliers (*n* = 44 private, *n* = 30 public patients). Same day admissions receive a value of LOS = 1

**Table 3:** Hospital acquired complications (HACs) in the index admission in the propensity-matched cohorts according to private health insurance status (New South Wales, 2017–2020)

HAC type	Private patients (*n* = 5315) n (%)	Public patients (*n* = 5315) n (%)	*p*-value
Cardiac complications	121 (2.28)	130 (2.45)	0.609
Healthcare associated infection	123 (2.31)	117 (2.20)	0.744
Surgical complications requiring unplanned return to theatre	113 (2.13)	77 (1.45)	0.010
Delirium	69 (1.30)	47 (0.88)	0.050
Respiratory complications	53 (1.00)	59 (1.11)	0.635
Medication complications	32 (0.60)	27 (0.51)	0.602
Gastrointestinal bleeding	22 (0.41)	29 (0.55)	0.400
Endocrine complications	17 (0.31)	21 (0.40)	0.626
Renal failure	7 (0.13)	8 (0.15)	1.000
Venous thromboembolism	6 (0.11)	5 (0.09)	1.000
Pressure injury	6 (0.11)	< 5^a^	0.289
Incontinence	< 5^a^	< 5^a^	0.687
Falls	< 5^a^	< 5^a^	1.00

^a^Cell suppressed as < 5 events

Length of stay: After excluding 74 outliers (*n* = 44 private, *n* = 30 public patients), the average LOS was 5.2 (± 4.4) days [Median 4.0 (IQR 3.0–6.0) days]. Private patients had a similar average LOS than public patients (mean difference = -0.11, 95%CI -0.28, 0.05, *p*-value = 0.182) (Table 2.). There were no differences among groups after further adjusting the average LOS for the presence of HACs (Mean difference = -0.10, 95%CI -0.25, 0.06, *p*-value = 0.239).

In-hospital mortality: Overall, rates of in-hospital mortality was low (*n* = 229, 2.2%). However, private patients were less likely to die in-hospital than public patients (OR: 0.59, 95% CI: 0.45, 0.77) (Table 2.). This corresponded to a reduction of approximately 11 deaths per 1,000 people among private compared to public patients undergoing PCI procedures for AMI in public hospitals.

### Post-hoc analysis of in-hospital mortality

**Fig. 2:**
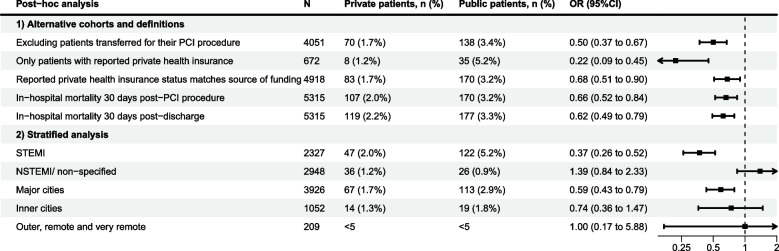
In-hospital mortality results from post-hoc analyses using matched cohorts. Note: Odds ratio < 1 indicates lower in-hospital mortality among private patients

Our post-hoc analyses all found results comparable with our primary analysis, with lower rates of mortality in private compared to public patients in all analyses using alternate cohorts and definitions (Fig. 2). When stratified by type of AMI (Fig. 2), significant differences in mortality outcomes were observed in STEMI patients only, with no significant difference in the subgroup of people with NSTEMI or non-specified AMI. When stratified by remoteness (Fig. 2), we observed significant differences in mortality outcomes only in major cities; however, we had small number of events in inner, outer, remote and very remote areas.

Finally, we estimated that the observed OR of 0.59, 95% CI: 0.45, 0.77 obtained in the primary analysis could be explained away by unmeasured confounders associated with both private health insurance and in-hospital mortality by an OR of 2.78 (95%CI 1.00, 3.87), adjusted for the currently measured covariates.

## Discussion

In this study of public hospital inpatients in Australia’s most populous state, we found privately insured AMI patients undergoing their first-observed PCI were approximately 40% less likely to die in hospital than publicly funded patients with similar health, socio-demographic, and hospital characteristics, representing a reduction of approximately 11 deaths/1,000 people undergoing PCI procedures for AMI in public hospitals. This difference was driven mostly by STEMI patients and those residing in major cities. However, we did not observe any difference between public and privately funded patients in rates of hospital acquired complications or length of stay. These seemingly contradictory findings suggest that private health insurance status may not be impacting in-hospital complications for AMI patients within Australia’s universal healthcare system but may still be associated with mortality outcomes, particularly among STEMI inpatients. This disparity likely reflects either further unmeasured differences between the types of public and privately funded patients who receive PCI, or potential differences in access to, and timing of, emergency services and treatment.

The profile of our study population [35–37] and our relatively low rates of in-hospital mortality [36–38], complications [36, 37], and average length of stay [6, 37] are broadly consistent with those from PCI populations reported in Australian jurisdictional or nationwide registries. Our findings by funding status are also largely consistent with international studies [12, 13]. For example, a US study of STEMI patients found a 35% difference in rates of in-hospital mortality, and no difference in average LOS, between privately insured and Medicaid patients [13]. However, there are few studies from Australia or other countries with universal health care arrangements with which we can directly compare disparities by health insurance status.

There may be multiple explanations for the disparity in mortality observed in our study. While we adjusted for a range of socio-demographic and health characteristics in our propensity scores, there may be further residual confounding in the types of private and publicly funded public hospital patients who receive PCI. For example, there may be differences in clinical presentation which could not be reliably measured using administrative data, such as salvage PCI, lesion characteristics, cardiogenic shock and cardiac arrest at presentation. Some of these characteristics reflect clinically unstable patients and are among the strongest predictors of in-hospital mortality, [12, 39] although it is not clear if there would be a differential prevalence relating to health insurance status. Similarly, the fact that disparities in mortality were seen in STEMI, and not NSTEMI patients, suggest these potential differences exist among the more severe AMI patients. The in-hospital mortality rate in our private STEMI PCI patients were much lower than the national average (4.6%), [38] suggesting these may be a healthier subset of PCI patients.

It is well known that a patients’ journey, from the onset of symptoms to the procedure, influences revascularisation outcomes, such as delays in first-medical contact and systems delays after presentation [4]. Patient care-seeking behaviours are associated with treatment delays, such as contacting primary health care providers (e.g., GPs) and not using emergency medical transport [40, 41]. In Australia, less than 15% of patients with AMI arrive in the emergency department within one hour of symptoms [40] (the optimal time window for reperfusion therapy), and the median time from symptoms to door is approximately 2.2 hours [40, 41]. Despite Australia having one of the shortest prehospital delays [41], ambulance services are not publicly funded—and uninsured people are potentially less likely to call for an ambulance while experiencing symptoms to avoid out-of-pockets costs for these services [40, 42, 43]. The relationship between private health insurance with other types of delay (e.g., time to electrocardiogram and diagnosis) is not well documented and worth exploring in future research. Those discrepancies may be particularly relevant for more severe forms of myocardial infarction (i.e., STEMI), as observed in our study. There may also be differences in the care provided during the hospital admission, such as the experience of the surgeon, the assessment of urgency of admission [44], use of fibrinolytic and other adjuvant therapy, and the type of intervention provided (e.g., bare metal or drug-eluting stenting), which we were unable to assess [13, 43].

Private health insurance is intrinsically tied to socioeconomic status, and there may be further demographic characteristics which we were not able to be identify (e.g. health literacy). Indeed, public patients in our study tended to be younger, more likely to be smokers, from lower socioeconomic areas, and with higher morbidities at presentation, suggesting a higher prevalence of marginalised populations with features that drive higher mortality. While our findings were consistent when restricting only to patients who reported holding private health insurance, it is still plausible there may be differences in the types of publicly and privately funded patients receiving a PCI. A recent US study found substantial differences in 30-day mortality between Medicare and Medicare Advantage patients admitted for AMI in 2009 had disappeared by 2018. These findings were unable to be explained, but hypothesised to reflect unmeasured changes in patients selected and enrolled in Medicare Advantage over time, as well as possible changes in coding practices [45]. We know that the clinical and demographic profile of patients receiving PCI has been changing in Australia over time, [6] and that there has also been differential uptake of elective PCIs in areas of high socioeconomic status [46]. Whether there are substantial differences in patient selection, clinical characteristics or hospital coding between private and public PCI patients in Australia is yet to be understood.

A key feature of Australian private health insurance is that it can increase or fast track access to ambulatory and specialist services [47], and it’s possible that the difference in mortality relates to service use prior to hospitalisation. Prior studies have reported differential access to cardiac services by socioeconomic factors (e.g. education) or insurance status [4]. Those disparities include increased use of angiography [15] presenting directly to PCI-capable hospitals and reduced time to reperfusion [43]. Other studies assessing disparities by indigenous status also highlighted that lack of private health insurance contributed to lower access to angiography and revascularisation procedures, [48, 49] including a lower probability of being transferred to metropolitan hospitals for angiography [50]. Finally, we acknowledge disparities by insurance status may occur in cardiovascular post-discharge care and events [9, 10, 16, 17, 43] and further studies should also investigate potential disparities post-discharge.

The limitations of administrative data are well known. Our study used one of the most comprehensive sets of patient socio-demographic and clinical characteristics in studies exploring disparities in mortality by insurance status, [7, 10, 12–14] but there are further unmeasured confounders not captured in administrative data, as acknowledged above. While few studies have identified the cause of disparities between patient outcomes by insurance status, the strength of association of such a confounder would need to be large (OR = 2.78), suggesting multiple factors may be at play. Further studies linking registry-based data and electronic health records with administrative data could help overcoming this limitation. Finally, Australia’s funding arrangements for hospital care are unique [23], and our findings may not be generalisable to other universal health systems. Furthermore, the use of a propensity matched study means our analysis is restricted to a particular subset of public and privately funded PCI patients, and may not be a representative sample. However, the fact we found similar disparities in outcomes of cardiovascular care to those reported in other health systems suggests there are commonalities in the patient experience. This Australian experience can inform the debate for countries considering expansion of universal health care with supplementary private health insurance [20].

## Conclusion

Privately insured patients admitted for AMI in Australian public hospitals and undergoing their first-observed PCI had lower in-hospital mortality rates than comparable publicly funded patients, and similar rates of in-hospital complications and average LOS. These results suggest in-hospital care is not the main contributor to inequities in mortality by insurance status, and other broader socioeconomic factors relating to patient selection, pathways of care and risk of mortality should be explored. Further studies including more granular information on patient, clinical and care pathways, and in countries with different structures of health systems and access to care, are required to understand and to improve equity in PCI health outcomes.

### Supplementary Information

**Additional file 1:** **Supplementary Table S1.** Detailed list of codes and definitions. **Supplementary Table S2.** Hospital peer groups. **Supplementary Figure S1.** Love plot of absolute standardised differences of baseline covariates between private and public patients before and after propensity score matching.

## Data Availability

This study used data from the New South Wales Admitted Patient Data Collection linked to the Registry of Births, Deaths and Marriages (RBDM) Death Registrations, and restrictions apply under the Health Records and Information Privacy Act 2002. Data not included in the article or uploaded as supplementary information are available upon reasonable request conditional to the permission of the data custodian and approval from the New South Wales Population and Health Services Research Ethics Committee.

## Abbreviations

ACHI: Australian classification of health interventions
AMI: Acute myocardial infarction
APDC: Admitted patient data collection
CABG: Coronary artery bypass grafting
CHeReL: NSW Centre for health record linkage
CVD: Cardiovascular disease
ED: Emergency department
EDDC: Emergency department data collection
HAC: Hospital acquired complications
ICD-10-AM: International classification of diseases 10^th^ revision Australian modification
IQR: Interquartile range
IRSD: Index of relative socioeconomic disadvantage
LOS: Length of stay
NSTEMI: Non–ST-elevated myocardial infarction
NSW: New South Wales
PCI: Percutaneous coronary intervention
PR: Prevalence ratio
RBDM: Registry of births, deaths and marriages death registrations data
SD: Standard deviation
STEMI: ST-elevated myocardial infarction
