# Supplementary material for: In-hospital outcomes by insurance type among patients undergoing percutaneous coronary interventions for acute myocardial infarction in New South Wales public hospitals
Source: Int J Equity Health. 2023 Oct 23;22:226. doi: 10.1186/s12939-023-02030-1 (PMC10594777; doi:10.1186/s12939-023-02030-1)
Supplement: Supplementary file 1 — Additional file 1: Supplementary Table S1. Detailed list of codes and definitions. Supplementary Table S2. Hospital peer groups. Supplementary Figure S1. Love plot of absolute standardised differences of baseline covariates between private and public patients before and after propensity score matching. [file 12939_2023_2030_MOESM1_ESM.docx]

**Appendix**

**Supplementary Table S1.** Detailed list of codes and definitions

| **Variable** | **Description** |
| --- | --- |
| ***Procedures*** | ***Australian Classification of Health Interventions (ACHI) codes***^1^ |
| Percutaneous coronary interventions | 38300-00, 38303-00, 38306-00, 38306-01, 38306-02, 38309-00, 38312-00, 38312-01, 38315-00, 38318-00, 38318-01, 90218-00, 90218-01, 90218-02, 90218-03  Multi-vessel PCI: 38315-00, 38318-00, 38318-01, 90218-01, 90218-03, 38303-00, 38306-02, 35305-00, 35310-02, 35305-00, 35341-00, 35344-00, 35310-02, 35344-01  Multi-stent PCI: 38312-01, 38318-01, 38306-01, 38306-02, 35310-01, 35338-01, 35310-02, 35344-01, 35310-01, 35310-02 |
| Coronary artery bypass grafting | 38497-00, 38497-01, 38497-02, 38497-03, 38497-04, 38497-05, 38497-06, 38497-07, 38500-00, 38500-01, 38500-02, 38500-03, 38500-04, 38500-05, 38503-00, 38503-01, 38503-02, 38503-03, 38503-04, 38503-05, 90201-00, 90201-01, 90201-02, 90201-03 |
| ***Medical history*** | ***International Statistical Classification of Diseases and Related Health Problems, 10th Revision, Australian Modification (ICD-10-AM) codes and definitions*** |
| Acute myocardial infarction | I21 as a principal diagnosis at index admission  Classification of AMI type:   - ST-elevated myocardial infarction – STEMI: I21.0–I21.3 ^1^ - Non–ST-elevated myocardial infarction – NSTEMI I21.4 ^1^ - Unspecified myocardial infarction: I21.9 ^1^ |
| Current smoking | F17.2, Z72.0 at index admission or in the prior 60 months ^2^ |
| Prior stroke | I63, I64 in the prior 12 months |
| Prior acute myocardial infarction | I21 in the prior 12 months |
| Charlson comorbidity score | Codes according to Sundararajan et al. (2004) ^3^ and supplementary codes for chronic conditions^5^ within the index admissions or in the prior 12 months |
| Hypertension  Coagulopathy | ICD-10 I10, I11–I13, I15 ^4^ or U82.3 ^5^ within the index admissions or in the prior 12 months  ICD-10 D65–D68, D69.1, D69.3–D69.6 ^4^ within the index admissions or in the prior 12 months |
| Prior hospital admissions | Number of hospital admissions in the prior 12 months, including at public or private hospitals |
| Prior ED presentations | Number of emergency department presentations at public hospitals in the prior 3 and 12 months |
| ***Admission characteristics*** | |
| Emergency status on admission | Emergency or elective admission status on first episode of the hospital stay |
| Emergency department mode of arrival | Mode of arrival to public emergency departments of current admission identified using the date of admission and date of ED presentation (with 1 day grace period). We classified the mode of arrive as emergency services (State ambulance, internal ambulance, helicopter rescue or air ambulance service), other (e.g., community/ public transport, private vehicle, no transport) or Unknown/NA. |

Sources:

^1^ Australian Institute of Health and Welfare (2021). Better Cardiac Care measures for Aboriginal and Torres Strait Islander people: sixth national report 2021. Canberra: AIHW.

^2^ Havard A, Jorm LR, Lujic S (2014). PLoS ONE 9(4): e95029. doi:10.1371/journal.pone.0095029

^3^ Sundararajan V, Henderson T, Perry C, Muggivan A, Quan H, Ghali WA. Journal of clinical epidemiology. 2004;57(12):1288-94

^4^ Quan H, Sundararajan V, Halfon P, Fong A, Burnand B, Luthi JC, et al. Medical care. 2005;43(11):1130-9

^5^ Australian Coding Standard (ACS) 0003 Supplementary codes for chronic conditions (<https://www.ihacpa.gov.au/>) were included to account for coding standards changes in 2015 in Australia

**Supplementary Table S2.** Hospital peer groups

| **Peer group** | **Description** |
| --- | --- |
| Principal referral | Greater than 35,000 acute weighted separations AND offering highly specialised services (such as bone marrow and other specialised transplants, severe burn injury, major trauma) |
| Major hospitals | 35,000 or less but greater than 10,000-17,000 acute weighted separations  Also considers the availability of one or more specialist services requiring specific infrastructure (such as cardiac catheterisation, comprehensive cancer centre, in-centre dialysis and medical radiation imaging) OR average acute NWAU per separation |
| District group | 4,000-10,000 or less but greater than 2,000 acute separations |
| Community hospitals | 2,000 or less acute separations.  Also considers total separations and percentual surgery |
| Other peer groups | Paediatric specialist, Ungrouped acute, Psychiatric, Nursing home, Multi-purpose service, Sub-acute, Palliative care, Rehabilitation, Mothercraft, Other ungrouped, Dialysis services |

Source: Adapted from NSW Hospitals 2014 Peer Group Classification


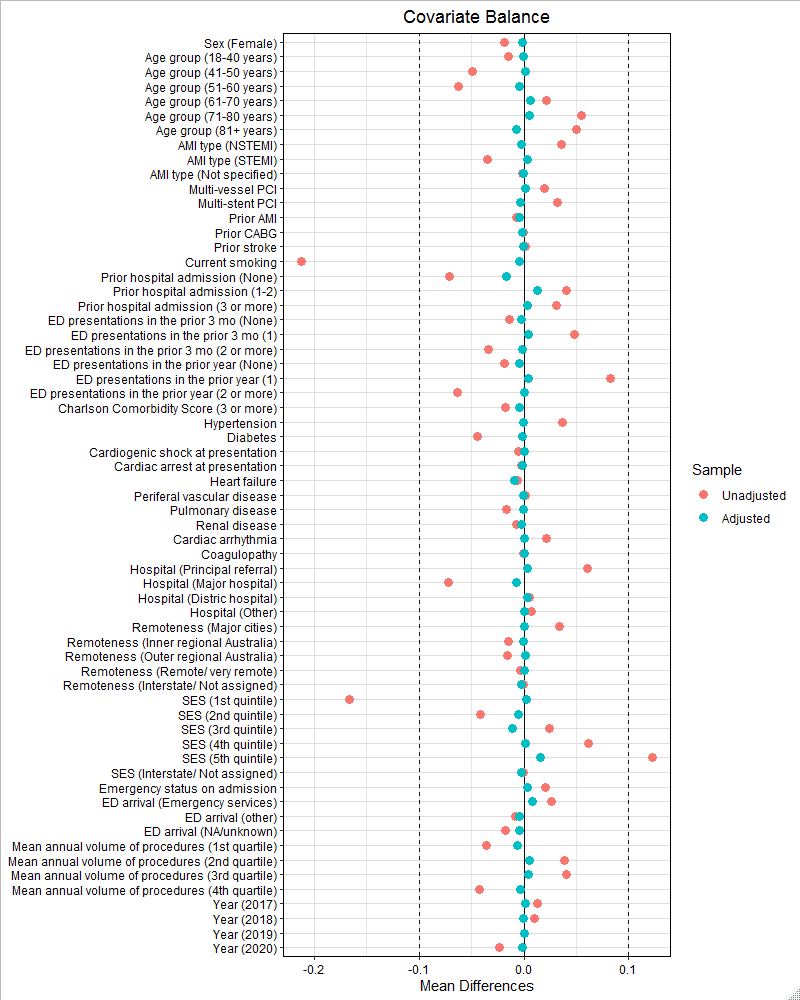


**Supplementary Figure S1. Love plot of absolute standardised differences of baseline covariates between private and public patients before and after propensity score matching.**

CABG: Coronary artery bypass grafting; AMI: Acute myocardial infarction; NSTEMI: non–ST-elevated myocardial infarction, PCI: Percutaneous coronary intervention; SES: Socioeconomic status; STEMI: ST-elevated myocardial infarction.
